# A retrospective, matched cohort study of potential drug-drug interaction prevalence and opioid utilization in a diabetic peripheral neuropathy population initiated on pregabalin or duloxetine

**DOI:** 10.1186/s12913-015-0829-9

**Published:** 2015-04-15

**Authors:** Jeffrey J Ellis, Alesia B Sadosky, Laura L Ten Eyck, Pallavi Mudumby, Joseph C Cappelleri, Lilian Ndehi, Brandon T Suehs, Bruce Parsons

**Affiliations:** Comprehensive Health Insights Inc., 325 West Main Street WFP6W, Louisville, KY 40202 USA; Pfizer Inc., 235 East 42nd Street, NewYork, NY 10017 USA; Formerly of Comprehensive Health Insights Inc., 325 West Main Street WFP6W, Louisville, KY 40202 USA; Humana Inc., 323 West Main Street WFP-05C, Louisville, KY 40202 USA

**Keywords:** Healthcare costs, Healthcare utilization, Medicare, Morphine equivalents, Drug-drug interaction, Pregabalin, Duloxetine

## Abstract

**Background:**

Anticipating and controlling drug-drug interactions (DDIs) in older patients with painful diabetic peripheral neuropaty (pDPN) presents a significant challenge to providers. The purpose of this study was to examine the impact of newly initiated pregabalin or duloxetine treatment on Medicare Advantage Prescription Drug (MAPD) plan pDPN patients’ encounters with potential drug-drug interactions, the healthcare cost and utilization consequences of those interactions, and opioid utilization.

**Methods:**

Study subjects required a pregabalin or duloxetine pharmacy claim between 07/01/2008-06/30/2012 (index event), ≥1 inpatient or ≥2 outpatient medical claims with pDPN diagnosis between 01/01/2008-12/31/2012, and ≥12 months pre- and ≥6 post-index enrollment. Propensity score matching was used to balance the pregabalin and duloxetine cohorts on pre-index demographics and comorbidities. Potential DDIs were defined by Micromedex 2.0 and identified by prescription claims. Six-month post-index healthcare utilization (HCU) and costs were calculated using pharmacy and medical claims.

**Results:**

No significant differences in pre-index demographics or comorbidities were found between pregabalin subjects (n = 446) and duloxetine subjects (n = 446). Potential DDI prevalence was significantly greater (p < 0.0001) among duoxetine subjects (56.7%) than among pregabalin subjects (2.9%). There were no significant differences in HCU or costs between pregablin subjects with and without a potential DDI. By contrast, duloxetine subjects with a potential DDI had higher mean all-cause costs ($13,908 vs. $9,830; p = 0.001), more subjects with ≥1 inpatient visits (35.6% vs 25.4%; p = 0.02), and more subjects with ≥1 emergency room visits (32.8% vs. 20.7%; p = 0.005) in comparison to duloxetine subjects without a potential DDI. There was a trend toward a difference between pregabalin and duloxetine subjects in their respective pre-versus-post differences in milligrams (mg) of morphine equivalents/30 days used (60.2 mg and 176.9 mg, respectively; p = 0.058).

**Conclusion:**

The significantly higher prevalence of potential DDIs and potential cost impact found in pDPN duloxetine users, relative to pregabalin users, underscore the importance of considering DDIs when selecting a treatment.

## Background

Challenging to patients, as well as providers, diabetes has the potential to cause a specific group of nerve disorders categorized as autonomic, peripheral proximal, focal, and peripheral neuropathies [1,2]. A common complication of diabetes, painful diabetic peripheral neuropathy (pDPN) affects approximately 1 in 4 people with this disease. Reduced glycemic control, advanced age of the patient, and the length of time the patient has diabetes are all documented risk factors for developing pDPN [3-6]. Diagnosis of the condition involves documentation of the symptoms of peripheral nerve dysfunction after exclusion of other potential causes of pain. The development of pDPN has been linked to signaling impairment in the central nervous system, although the process driving this change remains inadequately understood [7,8].

While the advancement of pDPN has been shown to be decreased through glycemic control [5], a variety of treatment options are available for pDPN; however, only a limited number have been proven effective in appropriate clinical trial settings [9,10]. There are a number of drug classes recommended and available including anticonvulsants, tricyclic antidepressants, and serotonin-norepinephrine reuptake inhibitors (SNRIs) [9,11]. Guidelines are available for treatment of pDPN; however, these can differ, and the recommended medications may generate adverse effects in conjunction with diabetic treatments [11,12]. Only three drugs, pregabalin, duloxetine, as well as the recently approved tapentadol, have been approved by the Food and Drug Administration for treatment of pDPN [13-20]. Evidence has been put forth for the use of opioids such as oxycodone and tramadol in treatment of pDPN through randomized controlled trials, although there is no long-term evidence of their impact on the disease or on quality of life of patients [9,11,21-24]. Stemming from the variable course of pDPN, the risk of opioid addiction in this group is increased, and a number of side effects are associated with opioids including hypogonadism and lowered immunity [25-27]. Consequences of opioid overdosing, especially in older patients, can be severe and clinical decision-making must balance the risk of such adverse effects by achieving pain control at the lowest possible combined dose(s) of opioid [28]. Previous retrospective studies across various patient populations and utilizing disparate definitions of opioid utilization have incongruent findings regarding the opioid-sparing effects of pregabalin [29-32] and duloxetine [29,30,33]. While clarity on the opioid-sparing effect of pregabalin and duloxetine should continue to be sought, it must be done so in light of introducing potential drug-drug interactions (DDIs) through use of either agent.

As the occurrence of pDPN increases with the degree of hyperglycemia and age of the patient, the total number of prescriptions a patient may be taking has the potential to rise dramatically due to the need to manage the effects of advancing diabetes and comorbidities related to the disease. As such, anticipating and controlling DDIs in patients with pDPN presents a significant challenge to providers, particularly in an older population. The consequences of these drug interactions have the potential to impact the quality of life of the patient, as well as increase the cost of the disease [9,34].

While some studies have examined aspects of treatment outcomes such as healthcare utilization and economic comparisons between pregabalin and duloxetine (where the costs were found to be comparable between the two drugs), few studies have documented the costs incurred from DDIs related to treatment of patients with pDPN [29,34-36]. Recently, Johnston et al. [34] examined the frequency and financial impact of DDIs and drug-condition interactions (DCIs) in predominantly commercially-insured pDPN patients prescribed pregabalin or duloxetine and found patients taking duloxetine had a substantially higher potential for DDIs and DCIs than patients taking pregabalin. Furthermore, the costs associated with potential DDIs and DCIs in patients taking duloxetine were found to be significantly increased.

The present study continues and builds on previous research by examining the impact of newly initiated pregabalin or duloxetine treatment on Medicare Advantage Prescription Drug plan (MAPD) pDPN members’ encounters with potential drug-drug interactions, the healthcare cost and utilization consequences of those interactions, and opioid utilization.

## Methods

### Study design and subject selection

This study is a retrospective, matched cohort study using medical and pharmacy claims collected from a large MAPD health plan. Medical and pharmacy claims data were extracted for the period of time between January 1, 2007 and December 31, 2012, and these data were used to identify potential subjects, measure patient characteristics, and examine study outcomes. MAPD members diagnosed with DPN were identified as potential subjects for the analysis based on International Classification of Disease, 9th revision, Clinical Modification (ICD-9-CM) diagnosis data reported on medical claims.

Members receiving pregabalin (Lyrica®) or duloxetine (Cymbalta®) between January 1, 2008 and June 30, 2012 were identified based on pharmacy claims, and the first observed prescription claim during this period was assigned as the study index date. Tapentadol was not included as a comparator as the study period did not allow for sufficient sample size to analyze members prescribed that agent. Identified members were further required to have 12 months of continuous pre-index enrollment during which no prescription claim for duloxetine or pregabalin was observed.

In order to enhance the likelihood that the medication of interest was being used to treat pDPN, it was further required that a diagnosis of DPN be observed within 90 days of the index date. Subjects meeting any of the following criteria were excluded from the study: Age <18 or >89 years; continuous enrollment <6 months post-index; diagnosis of fibromyalgia (FM) at any time after the index date; diagnosis and/or procedure code indicative of transplant surgery, cancer, or pregnancy during study period; long-term care facility residence of ≥90 days during the study period; or, one or more prescription claims for the non-index comparator drug (eg, if index drug is duloxetine, then non-index comparator drug is pregabalin) 12 months prior to or on the index date. The research protocol was reviewed and approved by Shulman Associates IRB, an independent Institutional Review Board, prior to study initiation. A waiver of informed consent and a waiver of authorization to use protected health information (PHI) were granted.

### Baseline measures

The subject’s assignment to either the pregabalin cohort or the duloxetine cohort was determined by the medication filled on the subject’s index date. Age, gender, race/ethnicity, geographic region of residence, low income subsidy (LIS) status, and Medicare dual eligibility (DE) status were measured based on member enrollment data. The Deyo implementation of the Charlson comorbidity index score was calculated based on medical claims adjudicated (insurer payment determined) during the pre-index observation period [37,38]. In addition to the Charlson score, specific pain-related medical conditions of interest were identified based on ICD-9-CM codes reported on medical claims during the pre-index period.

Pre-index and post-index medication utilization were measured based on pharmacy claims adjudicated during their respective observation periods. Pain-related medication use patterns were measured for the following medication classes: opioids, non-opioid analgesics, antidepressants, anticonvulsants, anesthetics, muscle relaxants, and anxiolytics. Individual subjects were identified as medication users if they had one or more prescription claims for the specific medication during the observation period. Opioid use was further examined and categorized as long-acting, short-acting low potency, and short-acting high potency [39]. In addition, intensity of opioid use was determined through conversion to morphine equivalents (MEq) based on established conversion factors [39]. The opioid dose strength and quantity of fill for each adjudicated opioid prescription claim was converted to MEq, summed for the observation period, and then normalized to an average MEq per 30 days.

### Study outcomes

#### Drug-drug interaction status

Medications with high potential for drug-drug interactions (DDI) with duloxetine or pregabalin (Table 1) were identified based on a drug interaction report generated from Micromedex 2.0 (Truven Health Analytics Inc., Greenwood Village, CO). Medications with an interaction classified as contraindicated, major, and moderate were included as potentially interacting drugs of interest. Medications with an interaction classified as minor or unknown in the Micromedex 2.0 system were not included as potentially interacting medications. Prescription claims were used to identify subjects receiving medication(s) that potentially interact with the index medication.

**Table 1:** **Pregabalin and duloxetine drug-drug interactions**

**Severity**	**Brief description of potential harm due to DDI**	**Interacting drug**
Pregabalin		
Major	Reduced pregabalin effectiveness	naproxen, ketorolac
Duloxetine		
Contraindicated	CNS toxicity or serotonin syndrome	isocarboxazid, linezolid, procarbazine, rasagiline, selegiline, tranylcypromine
	Increased serum concentrations of interacting drug and risk of cardiac arrhythmia	thioridazine
	Increased risk of extrapyramidal reactions or neuroleptic malignant syndrome	metoclopramide
	Increased risk of serotonin syndrome or neuroleptic malignant syndrome-like reactions	methylene blue
Major	Increased interacting drug plasma level and Increased risk of QT prolongation	clozapine
	Increased risk of bleeding	antiplatelet agents, escitalopram
	Increased risk of serotonin syndrome	almotriptan, citalopram, cyclobenzaprine, desvenlafaxine, dextromethorphan, eletriptan, fluoxetine, fluvoxamine, frovatriptan, hydroxytryptophan, lithium, lorcaserin, methadone, milnacipran, naratriptan, paroxetine, rizatriptan, sertraline, sumatriptan, tapentadol, tramadol, trazodone, tryptophan, venlafaxine, zolmitriptan
	Increased risk of serotonin syndrome or neuroleptic malignant syndrome-like reactions	vilazodone
	Increased serum concentrations of interacting drugs and an Increased risk of cardiotoxicity	class 1C antiarrhythmic agents
Moderate	decreased plasma concentrations of the active metabolites of interacting drug	tamoxifen
	Increased duloxetine serum concentrations and risk of adverse effects	ciprofloxacin, clobazam, enoxacin, mirabegron, quinidine
	Increased exposere to interacting drug and potential toxicity	phenothiazines, tamsulosin, tricyclic antidepressants
	Increased risk of bleeding	acenocoumarol, dabigatran, dalteparin, danaparoid, desirudin, enoxaparin, fondaparinux, nonsteroidal anti-inflammatory agents, phenindione, phenprocoumon, tinzaparin, warfarin

Source: Micromedex 2.0 (Truven Health Analytics Inc., Greenwood Village, CO), accessed May 31, 2013. Drugs with primary use in the hospital setting (e.g., lepirudin) were excluded.

Subjects were flagged with a DDI if they met either of the following criteria: 1) a prescription claim for an interacting medication during the pre-index period and a corresponding days’ supply indicative of coverage that overlapped the index date; or, 2) an adjudicated prescription claim for an interacting medication filled within 30 days after the index date.

#### Healthcare resource utilization and costs

All-cause medical service utilization and costs were measured using medical claims data. Medical claim place of service was used to assign inpatient, emergency room, and outpatient utilization and costs. Physical therapy (PT) utilization was identified using medical claims where the Current Procedural Terminology (CPT) code billed indicated a physical therapy service was performed. Total pharmacy costs were defined as the sum of costs (plan-paid and member-paid) associated with adjudicated pharmacy claims. Total all-cause healthcare costs were defined as the sum of the respective total medical cost and total pharmacy cost components. All cost calculations included both member and plan paid components, and were adjusted to 2012 United States (US) dollars based on the medical care component of the Consumer Price Index. Post-index opioid utilization was determined both categorically and as MEq per 30 days, as previously described for the pre-index period.

### Analysis

Propensity score (PS) matching was utilized to address potential bias in this non-randomized, observational study [40,41]. In the PS matching, all baseline demographic characteristics and medical conditions of interest were included. The PS matching process, matching a duloxetine member to a pregabalin member, was based on the nearest neighbor approach, without replacement, using a caliper width of 0.005.

Diagnostic evaluation was considered to examine balance in pre-index covariates (from the propensity score model) between matched treatment groups. In order to account for the non-normal data distribution, continuous pre-index measures were compared between-groups using non-parametric Wilcoxon rank-sum tests. Chi-square tests were used to compare categorical pre-index data. Wilcoxon rank-sum test and chi-square tests were also performed for post-index outcomes [42].

Multivariable statistical models were applied to examine the relationship between DDI status and healthcare costs [43]. Specifically, a generalized linear mixed model (GLMM) with a gamma distribution and log link was fitted to model total healthcare costs. Several variables were entered into the model. They included index drug, DDI status, demographic characteristics, baseline medical conditions, and pre-index medication utilization. An “index drug*DDI status” covariate interaction term was also included in the model. Means and confidence intervals were derived from the models and then retransformed into dollar values through exponentiation of the adjusted least square (LS) means estimates. All comparisons of total all-cause healthcare costs between and within cohorts were assessed using Wald chi-square tests.

Differences in all-cause total healthcare costs were compared between the cohorts (i.e., by pregabalin/duloxetine and DDI status) via Difference-in-Differences (DID) analyses, using the model-based LS Means estimates and corresponding standard errors:

DID = mean of values in (DDI present minus DDI absent in the pregabalin cohort) minus mean of values in (DDI present minus DDI absent in the duloxetine cohort).

T-tests were performed to formally test the DID values for statistical significance.

Differences in pre-index and post-index opioid utilization and MEq were compared between pregabalin and duloxetine cohorts via DID analysis. The t-statistic for the opioid utilization DID analysis was derived from a GLMM with a logit link and binary distribution, controlling for baseline demographics, Deyo-Charlson comorbidity index, and pre- and post-index non-opioid pain medication utilization. The t-statistic for the MEq DID analysis was derived from a GLMM with an identity link and a Gaussian distribution, controlling for baseline demographics, Deyo-Charlson comorbidity index, and pre- and post-index non-opioid pain medication utilization. The MEq DID analysis covered opioid prescription claims from 6-months pre-index to 6-months post-index.

All data analyses were conducted using SAS version 9.3/SAS Enterprise Guide 5.1. The *a priori* alpha level for all inferential analyses was set at 0.05; all statistical tests were two-tailed.

## Results

### Patient characteristics

A total of 1,320 subjects met the study inclusion and exclusion criteria (pregabalin n = 570, duloxetine n = 750). After conducting the propensity score matching procedure, the analytic cohort consisted of 446 matched pairs (892 total study subjects). The lack of statistically significant differences between the matched cohorts on baseline demographics (see Table 2) or pre-index comorbidity measures (see Table 3) provides evidence to support that the matching process was successful. The predominantly older composition of the study cohorts was confirmed as both the pregabalin and duloxetine groups exhibited a mean age of 66.7 years. The percentage of subjects with a potential DDI was greater in the duloxetine group compared to the pregabalin group (56.7%, n = 253 vs. 2.9%, n = 13, p < 0.001). There were 17 potential DDIs involving pregabalin, all of which were of “Major” severity. There were 649 potential DDIs involving duloxetine, including 11 “Contraindicated” interactions and 453 “Major” interactions (Table 4).

**Table 2:** **Patient characteristics**

	**Pregabalin**		**Duloxetine**		
	***N*** **= 446**		***N*** **= 446**		
	***n***	**%**	**n**	**%**	P value*
Age, years (categorical)					0.1304
18-29	0	0.0	0	0.0	
30-39	7	1.6	1	0.2	
40-49	27	6.1	26	5.8	
50-59	73	16.4	86	19.3	
60-69	144	32.3	159	35.7	
70-79	150	33.6	126	28.3	
80-89	45	10.1	48	10.8	
Gender					0.5854
Male	185	41.5	177	39.7	
Female	261	58.5	269	60.3	
Race/Ethnicity					0.7265
White	363	81.4	353	79.1	
Black	58	13.0	69	15.5	
Hispanic	13	2.9	14	3.1	
Other	12	2.7	10	2.2	
Geographic Region					0.6759
Northeast	5	1.1	5	1.1	
Midwest	78	17.5	78	17.5	
South	338	75.5	333	74.7	
West	25	5.6	30	6.7	
Low Income Status	87	19.5	92	20.6	0.6814
Dual Eligibility Status	52	11.7	56	12.6	0.6759
	**Mean**	**SD**	**Mean**	**SD**	
Age, years (continuous)	66.7	10.7	66.7	10.4	0.5924
Propensity Score	0.47	0.14	0.47	0.14	0.9931

*All P values for categorical variables calculated using chi -square tests. All P values for continuous variables calculated using Wilcoxon rank-sum tests.

SD, Standard Deviation.

**Table 3:** **Pre-index comorbid conditions**

	**Pregabalin**		**Duloxetine**		
	***N*** **= 446**		***N*** **= 446**		
	***n***	**%**	***n***	**%**	P value*
Abdominal pain/cramping	106	23.8	107	24.0	0.9374
Anxiety disorder	57	12.8	57	12.8	1
Arthritis	186	41.7	198	44.4	0.4171
Back pain with neuropathic involvement	123	27.6	126	28.3	0.8228
Causalgia and other painful neuropathies	109	24.4	112	25.1	0.8160
Depression	77	17.3	71	15.9	0.5892
Diabetes	388	87.0	397	89.0	0.3537
Epilepsy	9	2.0	10	2.2	0.8166
Fatigue	133	29.8	136	30.5	0.8268
Irritable bowel syndrome	6	1.3	6	1.3	1
Migraine headache	9	2.0	10	2.2	0.8166
Muscle weakness	50	11.2	49	11.0	0.9151
Neck pain with neuropathic involvement	36	8.1	42	9.4	0.4770
Other back pain	165	37.0	168	37.7	0.8355
Other mental disorders	1	0.2	1	0.2	**
Other musculoskeletal pain	333	74.7	338	75.8	0.6982
Other neck pain (spinal)	57	12.8	63	14.1	0.5560
Sleep disorders	111	24.9	114	25.6	0.8171
Substance abuse or dependence	57	12.8	59	13.2	0.8422
Tension headache	5	1.1	4	0.9	**
Thinking/memory loss	12	2.7	8	1.8	0.3657
Trigeminal nerve disorders	2	0.4	0	0.0	**
	**Mean**	**SD**	**Mean**	**SD**	
Deyo-Charlson Comorbidity Index score	2.6	2.2	2.7	2.2	0.3058

*All P values for categorical variables calculated using chi-square tests. All P values for continuous variables calculated using Wilcoxon rank-sum tests.

**Expected Counts <5. Chi-square test may not be appropriate.

SD, Standard Deviation.

**Table 4:** **Observed pregabalin and duloxetine drug-drug interactions by severity and interacting drug**

Severity*	**Interacting drug**	**Number of interactions**
Pregabalin		
Major	naproxen	17
	Total Major for pregabalin	17
	Grand total for pregabalin	17
Duloxetine		
Contraindicated	metoclopramide	11
	Total Contraindicated for duloxetine	11
Major	tramadol	35
Major	trazodone	31
Major	cyclobenzaprine	31
Major	meloxicam	29
Major	citalopram	25
Major	clopidogrel	91
Major	methadone	21
Major	fluoxetine	25
Major	ibuprofen	22
Major	escitalopram	11
Major	sertraline	32
Major	diclofenac	18
Major	paroxetine	17
Major	celecoxib	9
Major	naproxen	9
Major	venlafaxine	12
Major	nabumetone	4
Major	etodolac	3
Major	piroxicam	3
Major	lithium	4
Major	indomethacin	3
Major	cilostazol	10
Major	sulindac	6
Major	propafenone	2
	Total Major for duloxetine	453
Moderate	amitriptyline	40
Moderate	warfarin	59
Moderate	promethazine	15
Moderate	ciprofloxacin	14
Moderate	tamsulosin	25
Moderate	doxepin	8
Moderate	nortriptyline	6
Moderate	prochlorperazine	3
Moderate	enoxaparin	9
Moderate	imipramine	1
Moderate	dabigatran	1
Moderate	butalbital	4
	Total Moderate for duloxetine	185
	Grand total for duloxetine	649

*Severity category according to Micromedex 2.0 (Truven Health Analytics Inc., Greenwood Village, CO), as of May 31, 2013.

### Healthcare resource utilization

There were no significant differences in any form of healthcare utilization between pregabalin subjects with and without a potential DDI (see Table 5). Compared to duloxetine subjects without a potential DDI, significantly greater percentages of duloxetine subjects with a potential DDI had at least one inpatient hospitalization and at least one emergency room (ER) visit. Similarly, duloxetine subjects with a potential DDI had significantly higher mean numbers of visits per member for all-cause inpatient hospitalizations, ER visits, and outpatient (non-physical therapy) visits compared to those without a potential DDI.

**Table 5:** **6-month post-index all-cause healthcare utilization stratified by drug-drug interaction status**

	**Pregabalin**			**Duloxetine**		
	**No**	**DDI**		**No**	**DDI**	
	**DDI present**	**present**		**DDI present**	**present**	
	**n = 433**	**n = 13**	***P*** value*	**n = 193**	**n = 253**	***P*** value*
All-Cause Inpatient Visits						
Members with Hospitalization	123 (28.4%)	1 (7.7%)	**	49 (25.4%)	90 (35.6%)	0.0214
Hospitalizations Per Member, Mean (SD)	0.9 (2.8)	0.1 (0.3)	0.0897	0.5 (1.9)	1.3 (3.9)	0.0059
All-Cause Emergency Room Visits						
Members with Visit	100 (23.1%)	3 (23.1%)	**	40 (20.7%)	83 (32.8%)	0.0047
Visits per Member, Mean (SD)	0.5 (1.2)	0.6 (1.2)	0.8068	0.4 (1.0)	0.7 (1.2)	0.0035
All-Cause Outpatient Visits (non-physical therapy)						
Members with Visit	427 (98.6%)	13 (100.0%)	**	190 (98.4%)	250 (98.8%)	**
Visits per Member, Mean (SD)	14.6 (12.6)	12.8 (9.1)	0.7728	15.2 (13.6)	17.3 (11.1)	0.0044
All-Cause Physical Therapy Visits						
Members with Visit	57 (13.2%)	2 (15.4%)	**	33 (17.1%)	32 (12.6%)	0.1869
Visits per Member, Mean (SD)	0.8 (3.1)	0.3 (0.9)	0.8733	1.0 (4.1)	0.9 (3.5)	0.2342

*All P values for categorical variables calculated using Chi Square tests. All P values for continuous variables calculated using Wilcoxon Rank-Sum tests.

**Expected Counts <5. Chi-square test may not be appropriate.

DDI, Drug-Drug Interaction.

SD, Standard Deviation.

### Healthcare costs

There were no significant differences in all-cause healthcare costs, or component costs (eg, pharmacy), between pregabalin subjects with and without a potential DDI (see Table 6). Conversely, duloxetine subjects with a potential DDI exhibited significantly greater costs in comparison to duloxetine subjects without a potential DDI. Among duloxetine subjects, all-cause total healthcare costs were significantly higher for those with a potential DDI than those without (means: $13,908 vs. $9,830; p = 0.001). The component healthcare costs of total medical, inpatient-related, ER-related, and total pharmacy costs were also significantly higher in duloxetine subjects with a potential DDI compared to those without.

**Table 6:** **6-month post-index all-cause healthcare costs and component costs stratified by drug-drug interaction status**

	**Pregabalin**			**Duloxetine**		
	**No DDI present**	**DDI present**	***P*** value*	**No DDI present**	**DDI present**	***P*** value*
	**n = 433**	**n = 13**		**n = 193**	**n = 253**	
All-Cause Total Healthcare Costs, Mean (SD)	11,085 (16,820)	8,470 (13,491)	0.2156	9,830 (14,351)	13,908 (20,254)	0.001
All-Cause Total Medical Costs, Mean (SD)	8,339 (16,20)	6,115 (13,461)	0.1835	7,550 (14,081)	10,311 (16,609)	0.0278
*Outpatient-Related (non-physical therapy), Mean (SD)*	2,819 (5,639)	1,944 (2,366)	0.7170	2,711 (4,392)	2,517 (3,999)	0.8396
*Physical Therapy-Related, Mean (SD)*	93 (385)	17.7 (64.0)	0.5499	98 (363)	103 (516)	0.2824
*Inpatient-Related, Mean (SD)*	4,082 (12,371)	3,722.5 (13,421.7)	0.1458	3,782 (12,713)	5,873 (13,965)	0.0201
*ER-Related, Mean (SD)*	248 (700)	217.6 (429.6)	0.9152	224 (675)	386 (900)	0.0061
All-Cause Total Pharmacy Costs, Mean (SD)	2,746 (3,213)	2,355.9 (2,686.5)	0.3120	2,280 (2,396)	3,597 (11,125)	<0.0001

Costs in US Dallars ($).

*All P values for categorical variables calculated using chi-square tests. All P values for continuous variables calculated using Wilcoxon rank-sum tests.

DDI, Drug-Drug Interaction; SD, Standard Deviation.

### Model-adjusted healthcare costs

No significant differences were found in model-adjusted mean all-cause healthcare costs for subjects with a potential DDI versus those subjects without a potential DDI in either the pregabalin cohort ($33,942 vs. $31,878, respectively; p = 0.8159) or the duloxetine cohort ($39,445 vs. $36,424, respectively; p = 0.4208) (see Table 7). In accordance with those findings, the DID analysis found no significant difference in all-cause healthcare costs associated with the presence of a potential DDI attributable to the index medication (p = 0.8950).

**Table 7:** Model-based* 6-month post-index all-cause total healthcare costs by index medication and DDI status and difference-in-difference comparison between pregabalin and duloxetine

	**All-cause healthcare costs**	
	**Mean**	**95% confidence interval**
Pregabalin		
No DDI present	$31,878	$9,545 - $106,461
DDI present	$33,942	$8,995 - $128,076
Difference**	$2,064	-
*P* value	0.8159	-
Duloxetine		
No DDI present	$36,424	$10,847 - $122,314
DDI present	$39,445	$11,904 - $130,702
Difference**	$3,021	-
*P* value	0.4208	-
Difference-in-difference comparison	$2,064 vs. $3,021	
Statistical difference†	p = 0.8950	

*GLMM model with gamma distribution and log link. Model-based means, standard errors, and confidence intervals were derived from model and transformed into dollar values through exponentiation. All p values are calculated using Wald chi square tests through LSMEANS option. Presence of potential DDI, Drug used (duloxetine versus pregabalin), DDI*Drug interaction term, baseline demographic characteristics, baseline comorbidities present, and pre-index medication utilization controlled for in GLM model.

**Represents the additional healthcare costs associated with the presence of a potential DDI.

† *t*-test, utilizing model estimates, comparing pregabalin difference (DDI minus No DDI) to duloxetine difference (DDI minus No DDI).

DDI, Drug-Drug Interaction.

CI, Confidence Interval.

### Opioid utilization and morphine equivalents

The DID analyses for the associations of index medication with opioid utilization and morphine equivalents utilization are described in Tables 8 and 9, respectively. There was a trend toward a significant difference between pregabalin and duloxetine subjects in their respective pre-versus-post differences in mean morphine equivalents per 30 days [60.2 milligramsn (mg) and 176.9 mg, respectively; p = 0.058]. The DID analysis of pre-index and post-index opioid utilization, as defined by one or more claims for long-acting, short-acting less potent, and short-acting more potent opioids, found no significant difference associated with the index medication.

**Table 8:** Opioid utilization* in pre- and post-index 6-month periods and difference-in-difference comparison between pregabalin and duloxetine

	**Long-acting opioids**	**Short-acting less potent opioids**	**Short-acting more potent opioids**
	**%**	**%**	**%**
Pregabalin			
Pre-index	7.8	51.6	15.7
Post-index	10.1	53.8	17.5
Difference	+2.2	+2.2	+1.8
Duloxetine			
Pre-index	7.0	49.6	16.6
Post-index	10.1	50.9	19.3
Difference	+3.1	+1.3	+2.7
Difference-in-difference comparison**	2.2 versus 3.1	2.2 versus 1.3	1.8 versus 2.7
Statistical Difference**	t = −1.08, p = 0.281	t = 0.23, p = 0.816	t = −0.28, p = 0.782

*Defined as one or more opioid prescription fills during period.

**Difference in Difference t-statistic derived from a generalized linear mixed model with a logit link and binary distribution adjusting for baseline demographics, Deyo-Charlson comorbidity index, and pre- and post-index non-opioid pain medication utilization.

**Table 9:** **Morphine equivalents utilization in pre- and post-index 6-month periods and difference-in-difference comparison between pregabalin and duloxetine**

	**Morphine equivalents milligrams/30 days**	
	**Mean**	**Standard deviation**
Pregabalin		
Pre-index	525.7	1,682.0
Post-index	585.9	1,539.8
Difference	60.2	899.2
Duloxetine		
Pre-index	442.6	1,208.0
Post-index	619.5	1,652.7
Difference	176.9	936.5
Difference-in-difference comparison*	60.2 vs. 176.9	
Statistical difference*	t = −1.90, p = 0.058	

*Difference in Difference t-statistic derived from a generalized linear mixed model with an identity link and a Gaussian distribution adjusting for baseline demographics, Deyo-Charlson comorbidity index, and pre- and post-index non-opioid pain medication utilization.

## Discussion

The present study adds to the limited understanding of potential DDI prevalence, as well as the healthcare cost and utilization consequences of those interactions, in a predominantly older MAPD patient population newly initiated on pregabalin or duloxetine for the treatment of pDPN.

This present study found that pDPN subjects initiating treatment with pregabalin were significantly less likely to encounter potential DDIs than patients initiating duloxetine. The magnitude of DDI prevalence for duloxetine patients observed (56.7%) is likely driven by factors inherent to the medication itself and by the population in which it was studied. Duloxetine undergoes hepatic metabolism by cytochrome P-450 (CYP450) isoenzymes 2D6 and 1A2. Further, it inhibits isoenzymes 2D6 and 1A2 and is highly plasma protein bound resulting in many potential DDIs with other medications [44]. Alternatively, pregabalin has minimal pharmacokinetic or pharmacodynamic interactions with other medications as signified in this study by the low prevalence of potential DDIs found (2.9%) [45]. The comorbidity burden and associated medication use of the matched pDPN pregabalin and duloxetine cohorts align with previous studies of pDPN patients [29,34-36] and it has been shown that pain treatment itself is an independent risk factor for exposure to potential DDIs [46,47].

A recent study by Johnston et al. found the frequency of potential contraindicated, major, and moderate DDIs in DPN patients newly initiated on duloxetine to be 3.9%, 36.9%, and 33.8%, respectively [34]. Conversely, no DPN patients newly initiated on pregabalin encountered a potential DDI of any severity. Methodological variations between the aforementioned study [34] and present study may explain the differences in reported potential DDI prevalence, with the most noteworthy variation being the insurance plan case-mix amongst the study populations. The former study [34] was conducted in a younger, predominantly commercially-insured population while the present study was conducted in a Medicare population with a mean age of 66.7 years for the matched cohorts. This observation highlights the age-related vulnerability to drug interactions experienced by older patients [48,49].

Previous research reported no statistically significant differences in post-initiation all-cause healthcare costs for pDPN patients newly initiated on pregabalin or duloxetine [29,36]. The present study endeavored to determine the impact of potential pregabalin and duloxetine DDIs on 6-month post-initiation costs. Among duloxetine subjects, mean all-cause total healthcare, total medical, inpatient-related, emergency room, and total pharmacy costs were significantly higher for those with a potential DDI than those without. Non-statistically significant cost increases were found between the pregabalin members with and without a potential DDI. These findings are substantiated by the Johnston et al. study that found the combined presence of potential DDIs and potential drug-condition interactions (DCIs) were associated with significantly higher all-cause healthcare costs in duloxetine users but not so in pregabalin users [34].

The non-significant findings of the within-pregabalin cohort comparisons and in the difference-in-difference analysis must be tempered by the small number of pregabalin subjects with a potential DDI (n = 13). The increase in costs associated with potential DDIs within the duloxetine cohort, however, cannot be overlooked. Prior studies of chronic pain conditions have found that CYP450 interactions between specific opioids (codeine, fentanyl, hydrocodone, methadone, oxycodone, and tramadol) and concomitant medications that serve as substrates or inhibitors of CYP450 isoenzymes 3A4 and 2D6, including duloxetine, result in higher costs and greater healthcare utilization [50-52].

A retrospective analysis of 170,086 patients using claims data from 2004 to 2008 found significantly higher mean total 6-month costs in chronic pain subjects with an exposure to a potential DDI compared to matched subjects without an exposure ($8165 vs. $7498, respectively; p < 0.01) [50]. This same study found that potential DDI exposed patients versus non-exposed patients experienced significantly more office visits (19.10 vs 18.29; p < 0.01), outpatient visits (6.71 vs 6.39; p < 0.01), emergency department visits (0.46 vs 0.43; p < 0.01), and inpatient hospitalizations (0.13 vs 0.12; p < 0.01) [50]. The large sample sizes allowed for detection of small differences, making the statistically significant findings difficult to interpret in terms of clinical relevance. The increases in healthcare utilization in the presence of potential DDI exposure is directionally consistent with the duloxetine cohort in the present study with regard to percent of patients with inpatient hospitalization and ER visits and mean numbers of visits per member for inpatient hospitalizations, ER visits, and non-physical therapy outpatient visits.

Changes in opioid utilization after initiation of pregabalin or duloxetine, regardless of potential DDI occurrence, were also assessed in the present study. No changes in pre- to post-initiation of long-acting opioid, short-acting opioid, strong opioid, weak opioid, or “any” opioid use were found for pregabalin or duloxetine, as defined by the percentage of patients with one or more pharmacy claims, in the lone head-to-head retrospective analysis of these drugs in a pDPN population [29]. However, when defined as the number of prescriptions filled by opioid users, both pregabalin and duloxetine patients experienced statistically significant increases in mean number of prescriptions filled for short-acting, weak, and “any” opioids [29]. Duloxetine patients also exhibited significantly greater post-index use of long-acting opioids [29]. The difference between the increases observed for pregabalin and duloxetine were not compared. As in the aforementioned study [29], the present study found no difference between pregabalin and duloxetine patients in their respective pre-versus-post differences in the percentage of members with ≥1 pharmacy claims for long-acting opioids, short-acting more potent opioids, or short-acting less potent opioids.

Given that opioids within the same category, such as “long-acting,” vary in terms of potency, the present study expanded on the aforementioned research [29] by assessing opioid use as a function of morphine equivalents. A robust difference-in-difference analysis of 6-month pre-index and 6-month post-index morphine equivalents used identified a trend toward greater mean morphine equivalents per 30-day post-index increase in use in the duloxetine cohort relative to the pregabalin cohort (176.9 mg vs. 60.2 mg, respectively; p = 0.058). As opioids vary not only in duration of action but also in potency, this finding, while not providing clarity on the opioid-sparing differences of pregabalin compared to duloxetine, does support the use of morphine equivalents as a preferred method of comparing changes in opioid utilization.

Several limitations must be noted for studies involving administrative claims data. Causal effect cannot be assigned given the observational nature of this study. The use of secondary data is limited by the threat of validity posed by inaccurate data within healthcare information technology systems as well as by differences in coding and documentation practices at the provider, region, or site-specific level. Actual patient behaviors regarding medication consumption cannot be assessed through paid pharmacy claims. Further, reliance on paid pharmacy claims for medication utilization does not capture the use of over-the-counter medications or remedies impactful to the disease state in question. Indirect costs were not considered as part of this study. A reduction in confounding was attempted through the use of propensity score matching and multivariate modeling. These approaches are limited by only the covariates included in the statistical models. Even with this attempt at bias reduction, residual confounding from included covariates can still occur and confounding from unknown or unmeasured covariates remains a possibility. The use of “potential” DDIs does not imply the actual occurrence of clinically-impactful DDIs, and therefore limits the interpretation and applicability of the findings. Lastly, given the focus on pDPN patients, the results of this study should not be considered generalizable to other FDA-approved indications or off-label uses.

## Conclusion

The results of this retrospective analysis suggest that the real-world prevalence of potential duloxetine DDIs in a MAPD pDPN population is substantially greater than that of potential pregabalin DDIs. While it is desirable to find infrequent DDIs, the low prevalence of potential pregabalin DDIs places limits on both the performance and sensitivity of within or between drug comparisons. From a clinical perspective, the importance of considering DDIs during treatment selection should be considered due to the significantly higher prevalence of potential DDIs found in pDPN duloxetine users.

## Abbreviations

DDI: Drug-drug interaction
CI: Confidence interval
CPT: Current Procedural Terminology
DCI: Drug-condition interaction
DE: Dual eligibility
DID: Difference-in-difference
ER: Emergency room
FM: Fibromyalgia
GLMM: Generalized linear mixed model
HCU: Healthcare utilization
ICD-9-CM: International Classification of Disease, 9th revision, Clinical Modification
LIS: Low income subsidy
LS: Least square
MAPD: Medicare Advantage Prescription Drug
MEq: Morphine equivalents
mg: Milligrams
pDPN: Painful diabetic peripheral neuropathy
PHI: Protected health information
PS: Propensity score
PT: Physical therapy
SD: Statndard deviation
SNRI: Serotonin-norepinephrine reuptake inhibitors
US: United States
